# Supplementary material for: Endogenous retroviruses and TDP-43 proteinopathy form a sustaining feedback driving intercellular spread of Drosophila neurodegeneration
Source: Nat Commun. 2023 Feb 21;14:966. doi: 10.1038/s41467-023-36649-z (PMC9944888; doi:10.1038/s41467-023-36649-z)
Supplement: Supplementary file 3 — Reporting Summary [file 41467_2023_36649_MOESM3_ESM.pdf]

nature portfolio

Corresponding author(s): Dubnau, Josh and Chang, Yung-Heng

Last updated by author(s): Jan 9, 2023

Reporting Summary

Nature Portfolio wishes to improve the reproducibility of the work that we publish. This form provides structure for consistency and transparency in reporting. For further information on Nature Portfolio policies, see our Editorial Policies and the Editorial Policy Checklist.

Statistics

For all statistical analyses, confirm that the following items are present in the figure legend, table legend, main text, or Methods section.

n/a	Confirmed	
☐	☒	The exact sample size (*n*) for each experimental group/condition, given as a discrete number and unit of measurement
☐	☒	A statement on whether measurements were taken from distinct samples or whether the same sample was measured repeatedly
☐	☒	The statistical test(s) used AND whether they are one- or two-sided<br>*Only common tests should be described solely by name; describe more complex techniques in the Methods section.*
☐	☒	A description of all covariates tested
☐	☒	A description of any assumptions or corrections, such as tests of normality and adjustment for multiple comparisons
☐	☒	A full description of the statistical parameters including central tendency (e.g. means) or other basic estimates (e.g. regression coefficient) AND variation (e.g. standard deviation) or associated estimates of uncertainty (e.g. confidence intervals)
☐	☒	For null hypothesis testing, the test statistic (e.g. *F*, *t*, *r*) with confidence intervals, effect sizes, degrees of freedom and *P* value noted<br>*Give P values as exact values whenever suitable.*
☒	☐	For Bayesian analysis, information on the choice of priors and Markov chain Monte Carlo settings
☒	☐	For hierarchical and complex designs, identification of the appropriate level for tests and full reporting of outcomes
☒	☐	Estimates of effect sizes (e.g. Cohen's *d*, Pearson's *r*), indicating how they were calculated

Our web collection on statistics for biologists contains articles on many of the points above.

Software and code

Policy information about availability of computer code

Data collection	Zeiss ZEN; Sapphire Biomolecular Imager (Azure Biosystems)
Data analysis	Sapphire Biomolecular Imager (Azure Biosystems); ImageJ (V1.53q); GraphPad Prism (v9.0)

For manuscripts utilizing custom algorithms or software that are central to the research but not yet described in published literature, software must be made available to editors and reviewers. We strongly encourage code deposition in a community repository (e.g. GitHub). See the Nature Portfolio guidelines for submitting code & software for further information.

Data

Policy information about availability of data

All manuscripts must include a data availability statement. This statement should provide the following information, where applicable:

- Accession codes, unique identifiers, or web links for publicly available datasets
- A description of any restrictions on data availability
- For clinical datasets or third party data, please ensure that the statement adheres to our policy

There are no large datasets that would require deposition into public repositories. All data are reported in the manuscript, with the exception of multiple independent confocal images. But we place no restrictions on data availability.

Human research participants

Policy information about studies involving human research participants and Sex and Gender in Research.

Reporting on sex and gender	not applicable
Population characteristics	not applicable
Recruitment	not applicable
Ethics oversight	not applicable

Note that full information on the approval of the study protocol must also be provided in the manuscript.

Field-specific reporting

Please select the one below that is the best fit for your research. If you are not sure, read the appropriate sections before making your selection.

[x] Life sciences [ ] Behavioural & social sciences [ ] Ecological, evolutionary & environmental sciences

For a reference copy of the document with all sections, see nature.com/documents/nr-reporting-summary-flat.pdf

Life sciences study design

All studies must disclose on these points even when the disclosure is negative.

Sample size	No sample size calculations were performed in advance. Rather, we used sample sizes that are significantly larger than typical for similar experiments. For example, PMID: 24464041, PMID: 33479240, PMID: 35927421 and PMID: 36517509
Data exclusions	No data were excluded from any sample set where the experiment worked technically. There were cases where (e.g.) an antibody failed to label any of the groups (even positive controls) because of technical error. In those cases, the entire experiment was excluded.
Replication	Each experiment was performed for all groups in parallel. Independent experiments were used to test for replication. Cell culture assays were replicated at least for three times (n>=3). Adult Drosophila brain samples (final N values) were collected from repeated experiments (n>=2). All reported in the figure legends.
Randomization	Experimental and control groups of animals with desired genotypes were randomly selected to go through experimental aging process and manipulated in parallel in the same incubators. Then, those desired animals were randomly dissected to collect their central brains for imaging specific molecular markers at specific time windows in parallel.
Blinding	Blinding was not possible in most cases. For example, with lifespans, the experimental groups begin dying very quickly compared to the control groups. With confocal microscopy, we typically see almost no signal in negative control groups (e.g. phospho-TDP-43). These factors would 'unblind' any observer. For this, we did not perform blinding through experiments and analysis.

Reporting for specific materials, systems and methods

We require information from authors about some types of materials, experimental systems and methods used in many studies. Here, indicate whether each material, system or method listed is relevant to your study. If you are not sure if a list item applies to your research, read the appropriate section before selecting a response.

Materials & experimental systems

n/a	Involved in the study	
[ ]	[x]	Antibodies
[ ]	[x]	Eukaryotic cell lines
[x]	[ ]	Palaeontology and archaeology
[ ]	[x]	Animals and other organisms
[x]	[ ]	Clinical data
[x]	[ ]	Dual use research of concern

Methods

n/a	Involved in the study	
[x]	[ ]	ChIP-seq
[x]	[ ]	Flow cytometry
[x]	[ ]	MRI-based neuroimaging

Antibodies

Antibodies used	mouse anti-Repo (Developmental Studies Hybridoma Bank 8D12), rat anti-Elav (Developmental Studies Hybridoma Bank 7E8A10),

	mouse anti-Elav (Developmental Studies Hybridoma Bank 9F8A9), mouse anti-Flag (SIGMA-ALDRICH F3165), rabbit anti-pTDP-43 (SIGMA-ALDRICH SAB4200223), rabbit anti-pTDP-43 (proteintech 22309-1-AP); rabbit anti-γH2Av (Rockland Immunochemicals 600-401-914); mouse anti-V5 (Thermo Fisher Scientific R960-25); rabbit anti-TDP-43 (proteintech 10782-2-AP); mouse anti-HERV-K-Env (AUSTRAL Biologicals HERM-1821-5); mouse anti-GFP (Thermo Fisher Scientific MA5-15256); rat anti-mCherry (1:500, Thermo Fisher Scientific M11217); rabbit anti-STMN2 (Novus NBP1-49461); donkey anti-mouse Alexa Fluor 488 (Jackson ImmunoResearch Laboratories, 715-545-151); donkey anti-rabbit Alexa Fluor 488 (Jackson ImmunoResearch Laboratories, 711-545-152); donkey anti-rat Alexa Fluor 594 (Jackson ImmunoResearch Laboratories, 712-585-153); donkey anti-mouse Alexa Fluor 647 (Jackson ImmunoResearch Laboratories, 715-605-151); donkey anti-rat DyLight 405 (Jackson ImmunoResearch Laboratories, 712-475-153); goat anti-mouse-HRP (Jackson ImmunoResearch Laboratories, 115-035-174); goat anti-rabbit-HRP (Jackson ImmunoResearch Laboratories, 111-035-144)
Validation	All antibodies used in this study were from commercial vendors and the validation is reported on the manufacturers' websites.<br>mouse anti-Repo (Developmental Studies Hybridoma Bank 8D12), https://dshb.biology.uiowa.edu/8D12-anti-Repo<br>rat anti-Elav (Developmental Studies Hybridoma Bank 7E8A10), https://dshb.biology.uiowa.edu/Rat-Elav-7E8A10-anti-elav<br>mouse anti-Elav (Developmental Studies Hybridoma Bank 9F8A9), https://dshb.biology.uiowa.edu/Elav-9F8A9<br>mouse anti-Flag (SIGMA-ALDRICH F3165), https://www.sigmaaldrich.com/US/en/product/sigma/f3165<br>rabbit anti-pTDP-43 (SIGMA-ALDRICH SAB4200223), https://www.sigmaaldrich.com/US/en/product/sigma/sab4200223<br>rabbit anti-pTDP-43 (proteintech 22309-1-AP), https://www.ptglab.com/products/phospho-409-410--TDP43-Antibody-22309-1-AP.htm<br>rabbit anti-TDP-43 (proteintech 10782-2-AP), https://www.ptglab.com/products/TARDBP-Antibody-10782-2-AP.htm<br>rabbit anti-γH2Av (Rockland Immunochemicals 600-401-914), https://www.rockland.com/categories/primary-antibodies/histone-h2avd-phosphos137-antibody-600-401-914/<br>mouse anti-V5 (Thermo Fisher Scientific R960-25), https://www.thermofisher.com/antibody/product/V5-Tag-Antibody-Monoclonal/R960-25<br>mouse anti-HERV-K-Env (AUSTRAL Biologicals HERM-1821-5), http://www.australbiologicals.com/index.php?what=catalog&id=339<br>mouse anti-GFP (Thermo Fisher Scientific MA5-15256), https://www.thermofisher.com/antibody/product/MA5-15256.html?ef_id=Cj0KCQjwgYSTBhDKARIsAB8Kuku5_v68LYx9_pMKbUDrKEMITr3iI0q8rN69JZ9TljbWdE6a__W8bJwaAhjwEALw_wcB:G:s&s_kwcid=AL!3652!3!459736943987!!!g!!&cid=bid_pca_aup_r01_co_cp1359_pjt0000_bid00000_0se_gaw_dy_pur_con&gclid=Cj0KCQjwgYSTBhDKARIsAB8Kuku5_v68LYx9_pMKbUDrKEMITr3iI0q8rN69JZ9TljbWdE6a__W8bJwaAhjwEALw_wcB<br>rat anti-mCherry (1:500, Thermo Fisher Scientific M11217), https://www.thermofisher.com/antibody/product/mCherry-Antibody-clone-16D7-Monoclonal/M11217<br>rabbit anti-STMN2 (Novus NBP1-49461), https://www.novusbio.com/products/stathmin-2-stmn2-antibody_nbp1-49461

Eukaryotic cell lines

Policy information about cell lines and Sex and Gender in Research

Cell line source(s)	Human SH-SY5Y (ATCC, CRL-2266); Drosophila S2 cells (Thermo Fisher Scientific, R69007).
Authentication	Cell lines were not authenticated.
Mycoplasma contamination	All used cell lines were tested negative for mycoplasma contamination.
Commonly misidentified lines (See ICLAC register)	No commonly misidentified cell lines were used in the study.

Animals and other research organisms

Policy information about studies involving animals; ARRIVE guidelines recommended for reporting animal research, and Sex and Gender in Research

Laboratory animals	Drosophila melanogaster were utilized for these experiments through the whole adult stage. SPG-Gal4 (R54C07-Gal4, #50472) and UAS-myr-RFP (#7119) were obtained from Bloomington Drosophila Stock Center. The hTDP-43-KI and UAS-RFP-hTDP-43 were generous gifts from Professor David Morton (Oregon Health & Science University) and Professor Jane Y. Wu (Northwestern University School of Medicine). The following stocks, UAS-GFP-IR, UAS-mdg4-IR (UAS-gypsy-IR), UAS-hTDP-43, tub-Gal80ts and UAS-mCD8-GFP were used in our previous studies. To prevent artifacts from genetic variation between groups, all strains used in this study were backcrossed to our laboratory wild-type strain, Canton-S derivative w1118 (isoCJ1), for at least five generations.
Wild animals	No wild animals were used in the study.
Reporting on sex	Male flies were chosen as the experimental subjects throughout the study
Field-collected samples	No field collected samples were used in the study.
Ethics oversight	none required.

Note that full information on the approval of the study protocol must also be provided in the manuscript.
